# Bexarotene therapy ameliorates behavioral deficits and induces functional and molecular changes in very-old Triple Transgenic Mice model of Alzheimer´s disease

**DOI:** 10.1371/journal.pone.0223578

**Published:** 2019-10-09

**Authors:** Jonathan Mauricio Muñoz-Cabrera, Adrián Gabriel Sandoval-Hernández, Andrea Niño, Tatiana Báez, Angie Bustos-Rangel, Gloria Patricia Cardona-Gómez, Alejandro Múnera, Gonzalo Arboleda

**Affiliations:** 1 Behavioral Neurophysiology Laboratory, School of Medicine, Universidad Nacional de Colombia, Bogotá, Colombia; 2 Grupo de Muerte Celular, Instituto de Genética, Universidad Nacional de Colombia, Bogotá, Colombia; 3 Área de Neurobiología Celular y Molecular, Grupo de Neurociencias de Antioquia, Facultad de Medicina, Universidad de Antioquia, Medellín, Colombia; Nathan S Kline Institute, UNITED STATES

## Abstract

**Introduction:**

Bexarotene, a retinoid X receptor agonist, improves cognition in murine models of Alzheimer’s disease (AD). This study evaluated the effects of bexarotene on pathological and electrophysiological changes in very old triple transgenic AD mice (3xTg-AD mice).

**Methods:**

24-month-old 3xTg-AD mice were treated with bexarotene (100 mg/kg/day for 30 days). The Morris water maze was used to evaluate spatial memory; immunofluorescence and confocal microscopy were used to evaluate pathological changes; and *in vivo* electrophysiological recordings were used to evaluate basal transmission and plasticity in the commissural CA3-CA1 pathway.

**Results:**

In addition to cognitive improvement, bexarotene-treated 3xTg-AD mice were found to have 1) reductions of astrogliosis and reactive microglia both in cortex and hippocampus; 2) increased ApoE expression restricted to CA1; 3) increased number of cells co-labeled with ApoE and NeuN; 4) recovery of NeuN expression, suggesting neuronal protection; and, 5) recovery of basal synaptic transmission and synaptic plasticity.

**Discussion:**

These results indicate that bexarotene-induced improvement in cognition is due to multiple changes that contribute to recovery of synaptic plasticity.

## Introduction

Alzheimer’s disease (AD) is the world’s most common cause of dementia. This neurodegenerative disorder is pathologically characterized by amyloid β (Aβ) and hyper-phosphorylated tau aggregates, which form amyloid plaques and neurofibrillary tangles, respectively [1]. These two abnormal protein aggregates have been shown to provoke neuronal dysfunction and impaired synaptic plasticity [2, 3]. The amyloid hypothesis postulates that Aβ accumulation is the main trigger of subsequent pathogenic events [4]. Aβ clearance is mediated by apolipoprotein E (ApoE), which facilitates not only Aβ enzymatic proteolysis [5] but also its transport across the blood-brain barrier by LRP1-binding. For this reason, potentiation of ApoE-mediated Aβ clearance has been suggested as a therapeutic approach for decreasing Aβ accumulation and improving cognitive function [6].

The retinoid X receptor (RXR) family, part of the nuclear receptor superfamily, has three main members: RXRα, RXRβ, and RXRγ. All RXRs are activated by 9-cis retinoic acid–their common natural agonist. Activated RXRs form either homodimers or heterodimers with Class-II orphan receptors or Class-I receptors (PPARs, LXRs, FXR, PXR, and CAR) and regulate transcription through direct interaction with gene promoters [7]. RXR heterodimers can be activated by specific ligands of either RXRs or one of its partners. When both receptors are activated, the result is a cooperative response [8].

Bexarotene is a synthetic RXR agonist that generated therapeutic effects in murine models for neurodegenerative disorders like Parkinson´s disease, Huntington disease, glaucoma and epilepsy [9–12]. The drug increased brain ApoE expression, but also to improved cognition and accelerate Aβ clearance [13]. Bexarotene, a synthetic RXR agonist, has been demonstrated not only to increase ApoE expression, but also to improve cognition and accelerate Aβ clearance in AD murine models [13]. Other reports have also shown that bexarotene, either alone or in combination with PPARs and LXRs agonists, improved cognition in AD murine models [2, 14, 15]. Bexarotene has been shown to increase the number of neuronal progenitor cells and enhance dendritic complexity in ApoE3 and ApoE4 mice [16]. More recently, bexarotene has been reported to prevent the deleterious effect of Aβ in cytoskeletal organization, thus facilitating neurite and axonal growth [17]. The precise roles of RXR activation in synaptic functioning and other neuronal physiology and its beneficial effects on the disease are still not completely understood. This paper correlates beneficial electrophysiological changes induced *in vivo* by bexarotene with cognitive amelioration and neuropathological recovery in a model of AD using elderly triple transgenic (3xTg-AD) mice.

## Materials and methods

### Ethical statement

All animal procedures were performed in accordance with the Animal Research Reporting *in vivo* Experiments (ARRIVE) guidelines [18] and the Guide for the Care and Use of Laboratory Animals, 8th edition, published by the National Institutes of Health (NIH) and fulfilled Colombian regulations (law 84/1989 and resolution 8430/1993). The Ethics Committee for Animal Experimentation of the Universidad de Antioquia in Medellín, Colombia and that of the Universidad Nacional de Colombia in Bogotá, Colombia approved all procedures.

### Animals

Twenty-four-month-old male and female triple-transgenic AD mice (3xTgAD), previously described in detail [19], and wild-type littermates (WT), supplied by the Sede de Investigación Universitaria (Universidad de Antioquia in Medellín, Colombia), were used as experimental subjects. Animals were housed in a sound-attenuated room in polycarbonate cages in groups of five, had free access to water and food during the whole experiment and were kept in controlled environmental conditions of 12-h light/dark cycle (lights on from 07:00 to 19:00), 20 ± 1 °C room temperature, and 50 ± 10% relative humidity. Appropriate care was taken to reduce the number of animals used and animal suffering. Eighteen 3xTg-AD and eight WT mice were used for behavioral and biochemical analyses. Eight 3xTg-AD and eight WT mice were used for electrophysiological experiments. Female and male animals were evenly distributed in the experimental groups.

All animals received either 100 mg/kg bexarotene or polyethylene glycol 400 (30% v/v) and polysorbate-20 (1% v/v in distilled water) administered via oral gavage each day for 30 days.

## Morris water maze

Hippocampal-dependent spatial memory was evaluated using the Morris water maze (MWM) paradigm. As described in detail previously [2], a cylindrical white pool, 100 cm in diameter and 54 cm in height, was filled with water previously opacified with non-toxic white gouache paint to a depth of 35 cm. The water was maintained at a constant temperature of 22 ± 2 °C. A circular platform 10-cm in diameter was located 1.5 cm below the surface of the water during spatial learning and 1 cm above the surface of the water during the visible platform session. Extra-maze visual cues around the pool remained in fixed positions in the room containing the water maze throughout the experiment.

Initial spatial training: The aim was to have the mice learn to find the platform in two sessions each day for five days. Each session consisted of four successive trials, and each trial began with the mice placed pseudo-randomly in one of four starting positions. Before the initial trial, the animals were trained to stay on the platform for 30 s. Forty-eight hours after the learning phase, the animals were tested for retention during a 90-s probe trial without the platform. During the probe trial, the latency for reaching the exact platform location was determined. To control any differences in visual-motor abilities and/or motivation between experimental groups, latencies for reaching the platform were evaluated with a visible platform in four trials at the end of the retention test. Behavior was recorded by an automated system (Viewpoint, Lyon, France).

Spatial reversal training: After the initial spatial learning task, a reversal learning protocol was followed to test all animals. During reversal learning, the hidden platform was moved to the nearest quadrant at the right. Similarly to the initial training, reversal learning training was conducted for two trials each day for two days.

## Immunofluorescence

Similarly as described in detail previously [2], after the behavioral training, the mice were deeply anesthetized with 87.5 mg/kg Ketamine/12.5 mg/kg Xylazine, exsanguinated and perfused with 4% paraformaldehyde in phosphate buffered saline (PBS). Their brains were then removed and transferred to a 30% sucrose solution. Brains were cut into 50-μm coronal sections with a vibratome (Leica VT 1000S, Leica, Nussloch, Germany) and stored at -20°C in anti-freezing medium for further processing. The coronal brain sections preserved in anti-freezing medium were washed three times with PBS, followed by reduction of auto-fluorescence with 50 mM NH_4_Cl for 10 min at room temperature. Tissue was permeabilized using 1% BSA with 0.3% Triton X-100 in 0.1 mM PBS for 1 hour and incubated with primary antibodies. These included anti-β-Amyloid, 1–16 Antibody 6E10 (SIG-39320; Signet/Covance, Dedham, MA), which recognizes human APP/Abeta; anti-Apolipoprotein E antibody [D6E10] (ab1906) 1:500, which had been raised against a peptide sequence to recognize polymorphic amino acid position 158 (Abcam, Cambridge, MA); anti-binding adaptor molecule 1 (Iba1) (1:1000, Wako Bioproducts, Richmond, VA Cat. #019–19741); anti-glial fibrillary acidic protein (GFAP) (Rabbit, 1:500; polyclonal antibody obtained by immunization with a preparation of full length human recombinant GFAP (Abcam, Cambridge, MA, Cat#:AB7260), anti-NeuN (Rabbit, 1:500 polyclonal antibody obtained by immunization with a recombinant fragment corresponding to Human NeuN aa 1–100 (N terminal) (Abcam, Cambridge, MA, Cat#:AB104225), Anti-Phospho-Tau (Ser202, Thr205) Monoclonal (AT8), Catalog # MN1020. (1:500; Thermo Scientific, Rockford, IL, USA). After overnight incubation at 4°C, three fluorescent-labeled secondary antibodies were used: Alexafluor 488 (1:2500; Invitrogen, Grand Island, NY), Alexafluor 568 (1:2500; Invitrogen, Grand Island, NY) and goat Anti-Rabbit IgG H&L (AMCA) (1:500; Abcam Cambridge, MA, Cat#: AB123435). Nuclei were stained with Hoescht 33258 (Invitrogen H-1398, Grand Island, NY). For lectin staining, *Lycospersicon esculentum*, a fluorescence labeling lectin, was used with secondary antibody solution (1:200 DyLight 488 *Lycospersicon esculentum* Vector Labs Cat. #DL-1174). Finally, slices were rinsed twice with distilled water and covered with glycerol jelly.

### Confocal analysis

Most images were captured under confocal microscopy using a 20x objective, but a 10x objective was used for Lectin (Nikon Eclipse C1 plus; Nikon Instruments Inc., Melville, NY, USA). For all analyses, slices were always selected from the same rostrocaudal location. Two consecutive sections from each mouse (n = 4 mice per treatment) from various regions of the hippocampus were analyzed using ImageJ software (National Institutes of Health, Bethesda, MD). The number and area of Aβ plaques were semi-quantitatively analyzed for each image captured using ImageJ software (National Institutes of Health, USA). Areas identified by thresholding as positive for 6E10 immunoreactivity were individually inspected to confirm that they were plaques. Individual mean area was averaged per treatment in each genotype group. The number of animals per group is indicated in figure legends. Statistical comparisons were performed by ANOVA and *post-hoc* testing, as indicated in figure legends.

### Western blot and ELISA

Following the pharmacological treatment, all animals received behavioral training. On the last day of behavior experiments, all animals were anesthetized using Ketamine/Xylazine, and brains were extracted and dissected manually. Similarly as described in detail previously [2], the cerebral cortex and the hippocampus of each mouse were homogenized using 300 μl of ice-cold RIPA buffer (Pierce Biotechnology; Rockford, IL, USA) which contained a complete protease inhibitor cocktail and a complete phosphatase inhibitor cocktail (Complete, PhosSTOP, Roche Applied Science, Mannheim Germany) in a homogenizer (PRO Scientific, Oxford, Connecticut, USA) at full speed for 20 s. The homogenate was sonicated at 30% output for 10 s, then centrifuged (12,500 g) at 4°C for 45 min in a microcentrifuge (Thermo Scientific, Rockford, IL, USA). Proteins were quantified with the BCA protein assay kit (Thermo Scientific, Rockford, IL, USA), and 20-μg protein samples were run in polyacrylamide gel at 100 V. After electrophoresis, proteins were transferred to a polyvinylidene difluoride (PVDF) membrane (Amersham Hybond^™^-P; GE Healthcare, Buckinghamshire, UK) and incubated in blocking buffer (5% powder milk in TBS-Tween 20) for 1 h at 48 °C until probed. The PVDF membrane was then incubated in 5 ml of monoclonal primary antibodies containing [1:1000 mouse anti-GFAP (Abcam, Cambridge, MA, Cat#:AB7260), 1:1000 mouse anti-ApoE (Abcam, Cambridge, MA, Cat#:AB1907) and 1:1000 mouse Anti-ABCA1 (Abcam, Cambridge, MA, Cat#:AB18180)] in blocking buffer for 2 h at room temperature or overnight at 4°C, washed 3 times in TBS-Tween (5 min/wash), incubated in peroxidase-conjugated secondary antibodies for 1 h (1:2000) and then washed 3 times with TBS-Tween. Binding antibodies were detected using the ECL system (Thermo Scientific, Rockford, IL, USA). ELISA for Aβ (x-40) and Aβ (x-42) was performed with a RIPA soluble Aβ42 fraction using a commercial kit (Covance, SIG-38954, SIG-38956, Princeton, NJ, USA) according to the manufacturer’s instructions.

### Electrophysiology

Under general anesthesia with 1.5 g/kg of 25% urethane (Sigma-Aldrich) and 10 mg/kg of 2% xylazine (Sigma-Aldrich), the subject was placed in a stereotaxic frame (SR-6R, Narishige Inc., Tokyo, Japan). The skull was exposed through a longitudinal fronto-occipital incision, and the dura mater was cut and removed. Two holes were drilled (S1 File) for recording in CA1 at stereotaxic coordinates of AP = -2.0 mm from the bregma and L = 1.5 mm, left [20]. Stimulation in contralateral CA3 was provided at stereotaxic coordinates AP = -2.0mm from the bregma and L = 2.0 mm, right [20].

A hydraulic micromanipulator (SM-25C, Narishige Inc., Tokyo, Japan) was used to lower a tungsten microelectrode (5 MΩ, Microprobes) 1.5 mm from the pial surface in order to record field excitatory postsynaptic potentials (fEPSP) in CA1. For stimulation in CA3, another micromanipulator (SM-25A, Narishige Inc., Tokyo, Japan) was used to lower a concentric bipolar stimulating electrode 2.0 mm from the pial surface. A ground electrode was placed into the neck musculature.

The activity recorded with the microelectrode in CA1 was magnified 100 times using an AC-coupled preamplifier (NEX-1, Biomedical Engineering, New York, USA). This signal was band-pass filtered (0.1 Hz and 10 kHz cut-off frequencies) and further amplified 20 times, resulting in total amplification of 2,000 times the original. CA1 field activity was then digitized using a DigiData 1200 analog to digital converter (Axon Instruments, Foster City, CA USA) with 10 kHz sampling frequency. It was stored for off-line analysis using Clampfit 8.0 (Axon Instruments, Foster City, CA USA).

Monophasic electrical pulses (duration = 100 μs) were delivered to CA3 at 0.33 Hz through an Isolator-11 stimulus isolation unit (Axon Instruments, Foster City, CA USA) controlled by a 9514 Plus pulse generator (Quantum Composers, Bozeman, MT USA). Stimulus intensity (100 to 400 μA) was adjusted to obtain stable and reliable responses in CA1; the final location of stimulation and recording electrodes was adjusted according to the CA1 fEPSP waveform.

After reaching stable fEPSP recording, the effect of stimulus intensity on response amplitude was studied. Intensity varied from 100 to 400 μA with 50- μA steps and was tested with 60 single electrical pulses of 100 μs duration with 30 s intervals between stimuli of a given intensity in CA3. Once the input-output (I/O) relationship of the preparation had been established, the stimulus intensity necessary for evoking ~50% (I_50_) of maximal response was selected for evaluating short- and long-term plasticity.

The effect of the inter-stimulus interval (ISI) between pairs of pulses (50, 75, 100, 200, 300 and 500 ms) on the ratio between the amplitude of the second response and that of the first response (R_2_/R_1_ ratio) was studied by delivering 60 pairs of electrical pulses (100-μs duration, 30-s inter-pair interval, I_50_ intensity) with a given ISI in CA3.

Baseline CA1 response to contralateral CA3 (cCA3) stimulation of 100-μs at I_50_ intensity and 0.1-Hz frequency was recorded for 10 min. Long-term potentiation of cCA3-to-CA1 synapses was induced by delivering six 1-s,100-Hz trains at 60-s intervals between trains (High-frequency stimulation, HFS). The effect of HFS on CA1-to-cCA3 synaptic efficiency was studied by recording the response to I_50_ cCA3 stimulation (100-μs duration, I_50_ intensity, 0.1-Hz frequency) for 60 min. Finally, the cCA3 stimulus intensity was increased from 100 to 400 μA in 50- μA steps, so that the effect on CA1 response amplitude could be studied again, as described above.

A deep level of anesthesia was maintained throughout the entire recording session by supplemental anesthetic doses about every 4 hours, achieving stable and reliable activity in CA1 (Zandieh et al., 2003).

Data were expressed as the percentage of change against baseline (mean ± S.E.M), using Clampfit software (V.8.0, Axon Instruments).

To verify the final location of electrodes at the end of the electrophysiological recordings, every brain was submerged in 4% paraformaldehyde for 3 days and were then cut in a vibratome (Leica VT 1000S, Leica, Nussloch, Germany) into coronal slices of 100 μm. Slices were examined in stereoscope (Olympus SZX16), and microphotographs were taken using a camera adapted for the purpose (Sony Cybershot DSCW7) (S1B and S1C File).

## Results

### Bexarotene treatment restores cognitive deficits in very old 3xTg-AD mice

**Fig 1:**
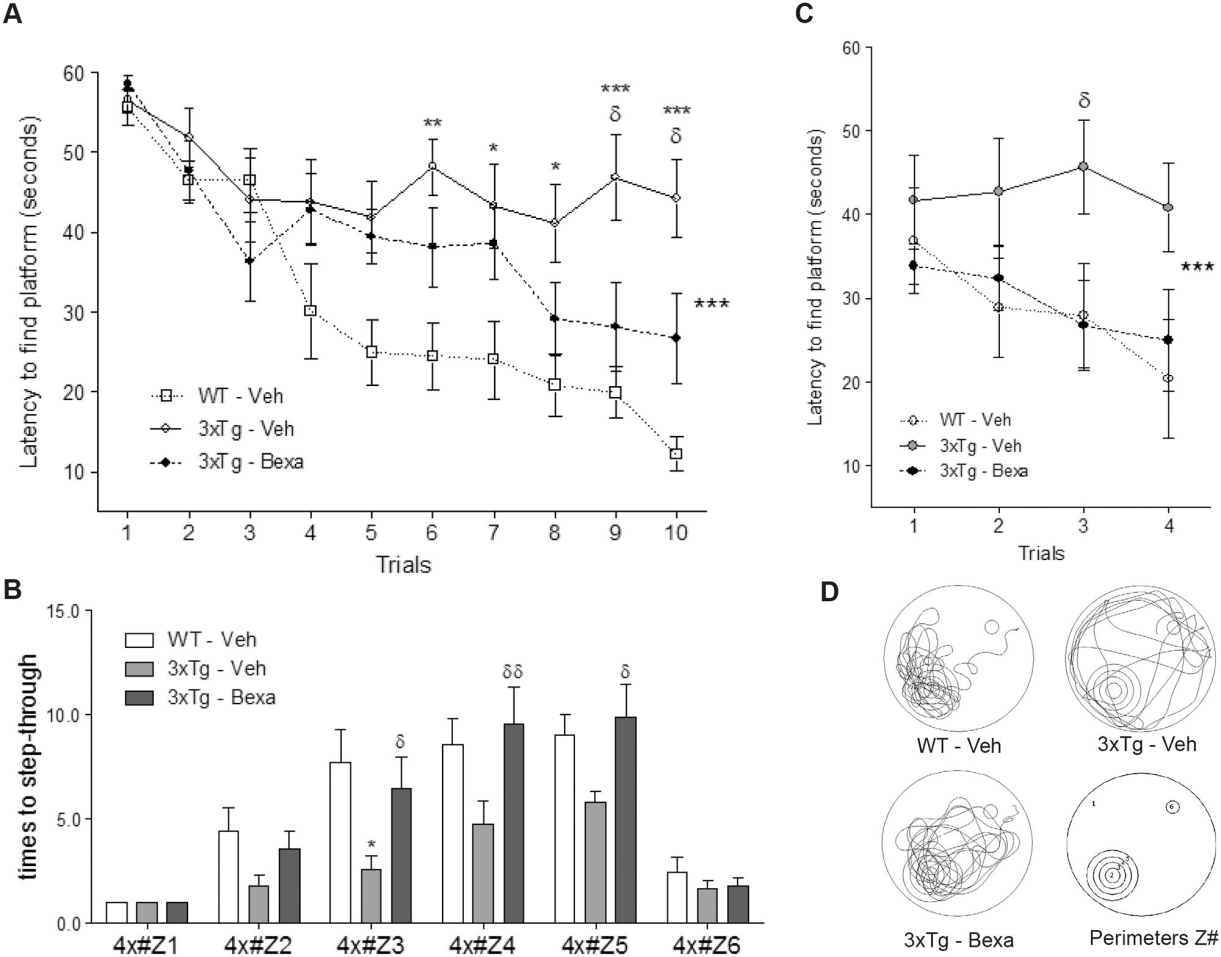
Bexarotene restores memory and cognition in very old 3xTg-AD mice. **(A)** Learning task: significant reduction in latencies to find the platform in treated 3xTg-AD mice against untreated 3xTg-AD mice over the five consecutive days. Statistically significant differences were found by using two-way ANOVA followed by Bonferroni posttest. *** p < 0.001. **(B)** Reversal learning: Bexarotene treated 3xTg-AD mice took significantly less time to find the new location of the platform after two consecutive days. **(C-D)** Retention tasks: **(C)** 4X analysis: times to step-through former platform location. **(D)** Samples of paths taken throughout the retention task exemplify preferences of treated and untreated WT and 3xTg-AD mice. All data were expressed as mean ± S.E.M. * represents p < 0.05 compared to WT; ^δ^ represents p < 0.05, ^δδ^ represents p < 0.01 compared to untreated 3xTg-AD mice. Cohort sizes were WT, n = 8; untreated 3xTg-AD mice, n = 9; treated 3xTg-AD mice (Randomized Females and Males), n = 9. Learning task: All data were expressed as mean ± S.E.M. * represents p < 0.05, ** represents p < 0.01, *** represents p < 0.001 compared to WT; ^δ^ represents p < 0.05 compared to untreated 3xTg-AD mice.

The effects of bexarotene activation of RXRs on cognition in 24-month old 3xTg-AD mice were evaluated using a spatial task in the Morris Water Maze (MWM). Untreated WT mice executed the task significantly better than untreated 3xTg-AD mice during the last five training trials of the learning task (p < 0.001) (Fig 1A). Bexarotene-treated 3xTg-AD mice’s performances were significantly better than those of untreated 3xTg-AD mice by the end of training (p < 0.01). The retention task was evaluated using a 4X design proximity analysis to count the number of times that mice stepped through an area near the platform (Fig 1B and 1D). The evaluation showed that bexarotene-treated 3xTg-AD mice stepped through the place where the platform had previously been located significantly more times than did untreated 3xTg-AD mice (p < 0.005). No significant differences were found between bexarotene-treated 3xTg-AD mice and WT mice. Spatial reversal training was also assessed, and the results were similar to those obtained for the learning task. Untreated WT mice performed significantly better than untreated 3xTg-AD mice during two days of trials. The data indicates that RXR activation improves hippocampal-dependent cognition tested by MWM in 3xTg-AD mice.

### Bexarotene reduces soluble β-amyloid but not β-amyloid plaques in the cortex of very old 3xTg-AD mice

**Fig 2:**
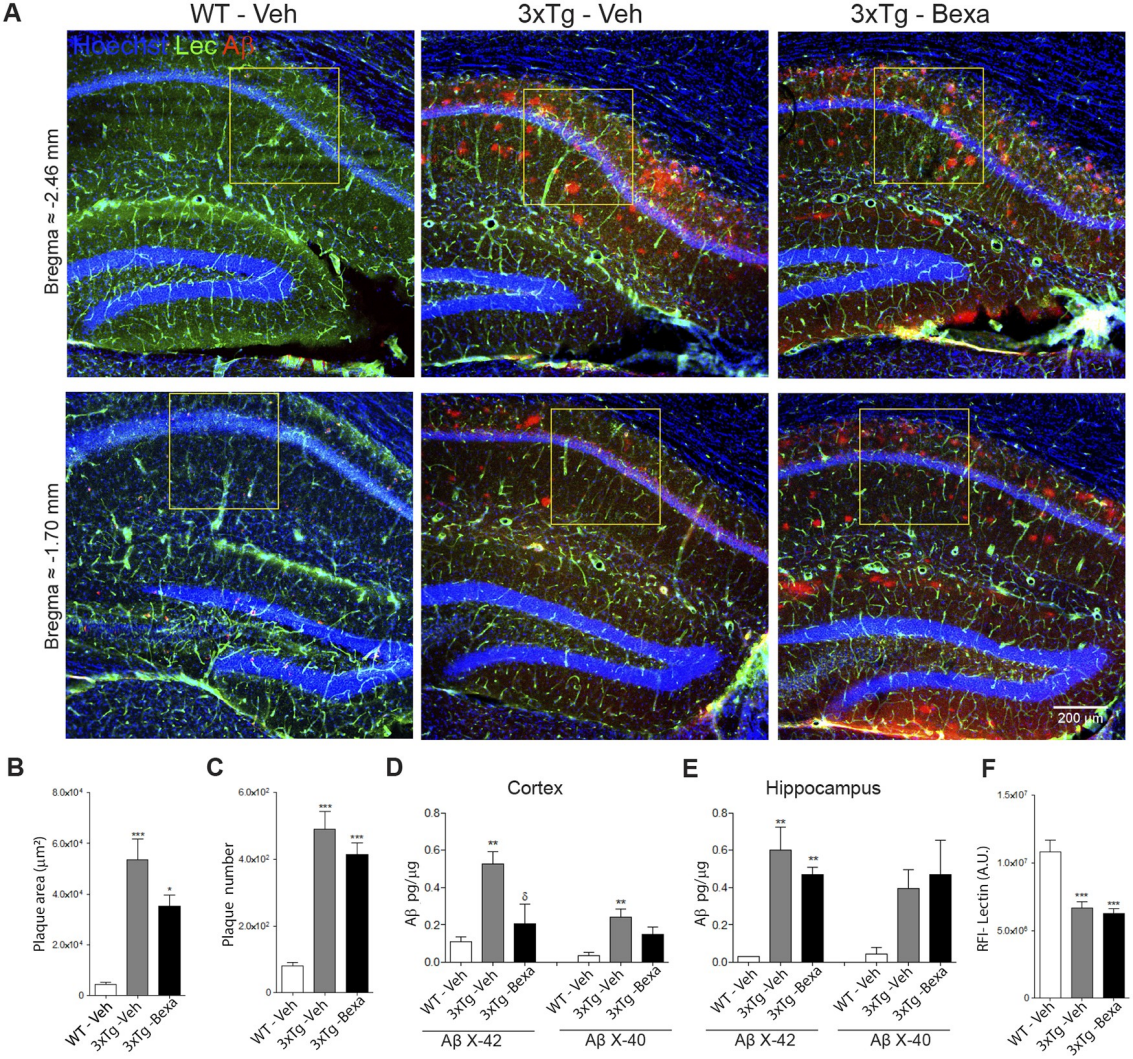
Bexarotene treatment did not affect amyloid pathology or vessels. Amyloid plaque deposition and vessels in brains were evaluated by immunofluorescence, and soluble Aβ was evaluated by ELISA. **(A)** Representative micrographs of hippocampus brain sections with Aβ immunofluorescence (Red) using anti-Aβ (6E10) antibody and lectin staining (Green) using Lycospersicon esculentum lectin and nuclei stained with Hoechst (Blue). **(B-C)** No significant differences were observed in the anti-Aβ positive area or number of plaques in the hippocampi of treated 3xTg-AD mice. Data were represented as mean ± S.E.M., Statistical analyses were carried out by one-way ANOVA followed by the Bonferroni test. Females (n = 4) per 3xTg-AD group. **(D-E)** The ELISA test were used to measure RIPA soluble Aβ42 and Aβ40. Data were represented as mean ± S.E.M. Statistical analyses made use of one-way ANOVA followed by the Bonferroni test. (n = 5) per group. **(F)** Relative fluorescence intensity (RFI) of Lectin in CA1 of the hippocampus (yellow box). Data are represented as mean ± S.E.M. Statistical analyses were performed by one-way ANOVA followed by the Bonferroni test. n = 5 per group.

The effects of bexarotene on clearance of amyloid burden have been controversial. First reports showed that Bexarotene accelerates reduction of amyloid plaques in 6-month old Tg2576 murine models of AD [13], but other reports showed that reduction varied depending on the model, laboratory, and pharmacological administration [21–25]. Immunofluorescence for human Aβ and ELISA for amyloid soluble forms were used to evaluate the effects of bexarotene on the relation of amyloid pathology to cognitive improvement. A higher density of Aβ plaques were found at the subiculum/CA1 boundary in 3xTg-AD mice while fewer plaques were found in the cortex. This is the same distribution of Aβ deposits previously reported for this age [26, 27]. No significant differences were found in number or area of amyloid plaques in the 3xTg-AD mice after bexarotene treatment (Fig 2A–2C), but a significant reduction (p < 0.05) in the RIPA soluble Aβ (1–42) fraction was found in the cortex (Fig 2D).

### Bexarotene up-regulates ApoE protein expression in CA1 of the hippocampus of very old 3xTg-AD mice

**Fig 3:**
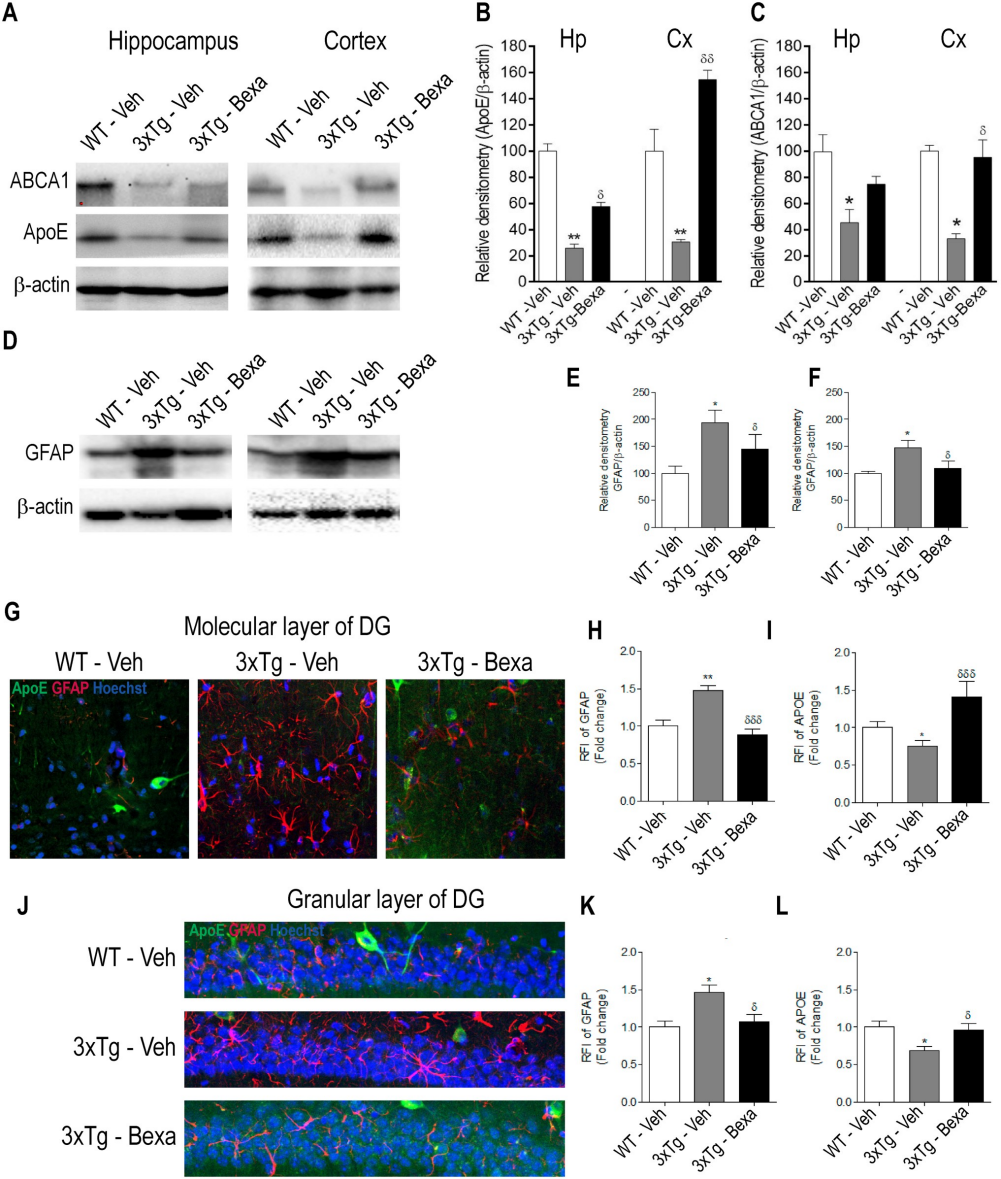
Bexarotene reduced astrogliosis and increases ApoE and ABCA1 in the hippocampus of treated 3xTg-AD mice. **(A)** Representative western blot of ApoE and ABCA1 expression. **(B-C)** Quantitative analysis of data presented in A showing changes in expression of (B) ApoE and (C) ABCA1. **(D)** Representative western blot of GFAP in hippocampus and cortex. **(E-F)** Quantitative analysis of data presented in A showing a significant reduction of GFAP expression in hippocampus and cortex of treated 3xTg-AD mice. **(G)** Representative immunofluorescence microphotographs of GFAP (Red), ApoE (Green) and Hoechst (Blue) in molecular layer CA1 of the hippocampus. **(H-I)** Quantitative analysis of data presented in G showing significant changes of RFI of **(H)** GFAP and **(I)** ApoE in treated 3xTg-AD mice. **(J)** Representative immunofluorescence microphotographs of GFAP (Red), ApoE (Green) and Hoechst (Blue) in granular layer of CA1 of the hippocampus. **(K-L)** Quantitative analysis of data presented in J, showing significant changes of RFI of **(K)** GFAP and **(L)** ApoE in treated 3xTg-AD mice. Statistical analyses was done by using one-way ANOVA followed by the Bonferroni test. Data are expressed as mean ± S.E.M. Differences against the control WT: *: p < 0.05, **: p < 0.01; and differences against the untreated 3xTg-AD mice: ^δ^: p < 0.05, ^δδ^: p < 0.01 ^δδδ^: p < 0.001; n = 4 per group.

Bexarotene increases ApoE expression rapidly in murine models of AD [13], and this has been associated with reduction of the amyloid plaque burden and improvement of cognitive function. Confocal microscopy analysis was used to determine if the amount of ApoE increases rapidly in the acute pathological condition of very old 3xTg-AD animals. Significantly less ApoE expression was found in untreated 3xTg-AD mice than in control WT animals (p < 0.001). ApoE expression in CA1 was significantly greater in bexarotene-treated 3xTg-AD mice than in untreated 3xTg-AD mice (p < 0.05), but this disparity was not found in the dentate gyrus (DG), CA3, entorhinal cortex (Ent-Cx) or neocortex (Cx) (Fig 3 and S1 File).

### Bexarotene reduces the amounts of reactive astrocytes and activated microglia markers in very old 3xTg-AD mice

Nuclear receptor agonist PPARs and LXRs have been reported to have anti-inflammatory effects on murine models of AD [13, 28, 29]. Astrocyte morphology was evaluated by immunofluorescence of the glial fibrillary acidic protein (GFAP) (Fig 3 and S2 File). Significantly higher levels of GFAP were found in CA1 (p < 0.05, n = 4), Cx (p < 0.01, n = 4) and Ent-Cx (p < 0.01, n = 4) of untreated 3xTg-AD mice than in CA1 of the control WT subjects (Fig 3), but no significant differences in the GFAP expression levels were found between bexarotene-treated 3xTg-AD mice and untreated 3xTg-AD mice in DG, CA3 (p < 0.01, n = 4), CA1 (p < 0.001, n = 4) and Cx and Ent-Cx (p < 0.001, n = 4) (Fig 3B).

**Fig 4:**
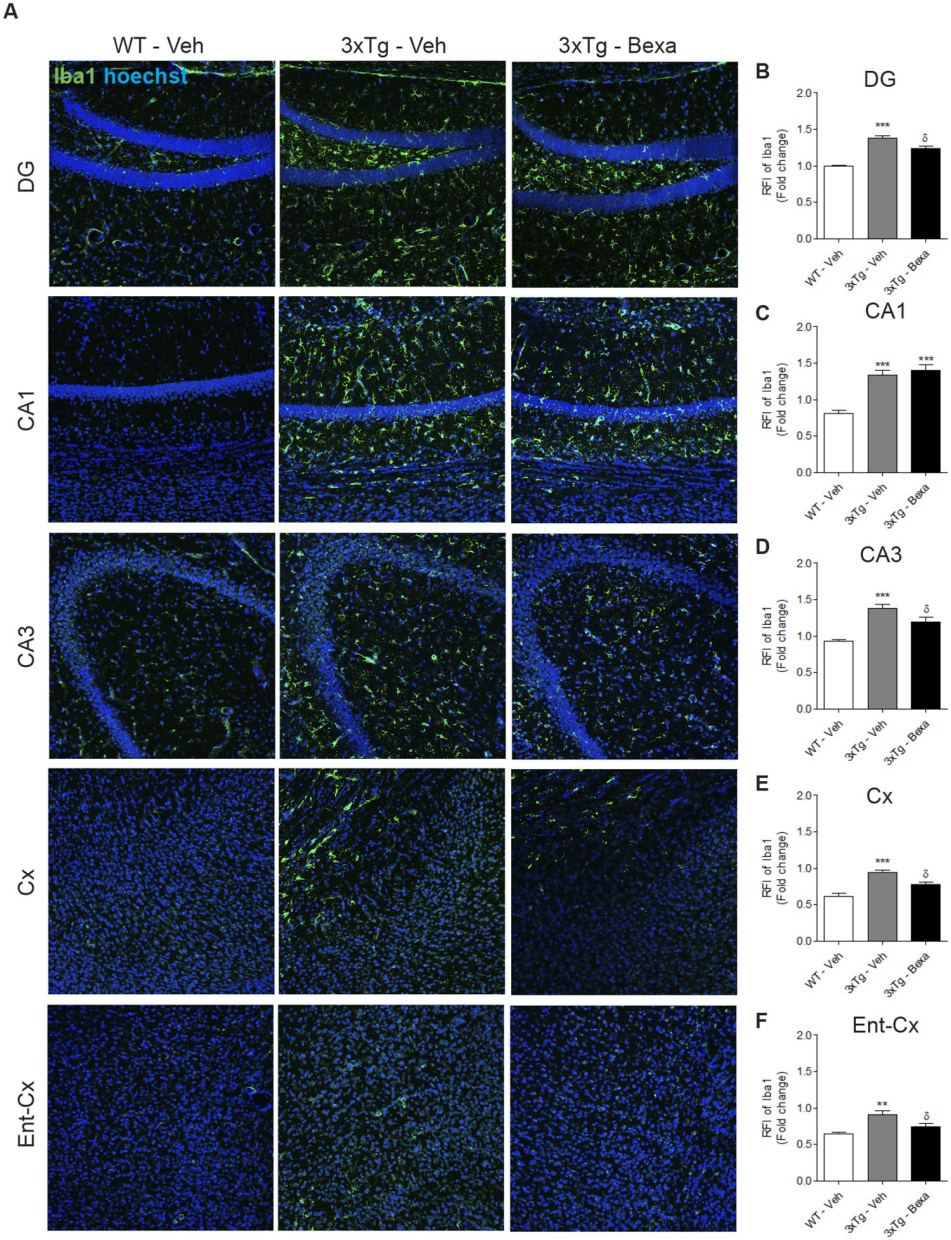
Bexarotene decreased Iba1 immunoreactivity in very old 3xTg-AD mice. **(A)** Representative micrographs of Iba1 immunofluorescence (Green) and Hoescht (Blue) using confocal microscopy. The hippocampus (DG, CA3 and CA1) Entorhinal cortex (Ent-Cx) and Neocortices (Cx) of untreated WT mice and treated and untreated 3xTg-AD mice were analyzed. **(B-F)** Quantitative analysis of Iba1 immunoreactivity in A. **(B)** DG. **(C)** CA1 of the hippocampus **(D)** CA3 of the hippocampus. **(E)** Cortex (Cx). **(F)** Entorhinal Cortex (Ent-Cx). Data were represented as mean ± S.E.M. Statistical analyses used one-way ANOVA followed by the Bonferroni test. * represents p < 0.05, ** represents p < 0.01 *** represents p < 0.001 compared with WT; ^δ^ represents p < 0.05 compared with untreated 3xTg-AD mice. n = 4 per group.

Morphological changes on GFAP+ and Iba1+ cells were also found. GFAP+ cells of untreated 3xTg-AD mice had less branches and thicker processes than did WT mice and, unlike the WT mice, they also had hypertrophic branches and soma (Fig 3). Bexarotene treatment partially reversed these morphological changes (Figs 3 and 4). Iba1 staining showed significantly greater intensities in the DG, CA1 and CA3 areas of the hippocampus and in the entorhinal cortex (Ent-Cx) of untreated 3xTg-AD mice than in those of the control WT animals (p < 0.01, p < 0.001, n = 4), but significantly less Iba1 staining was found in the DG, CA3, Cx and Ent-Cx of bexarotene-treated 3xTg-AD mice than in those areas in untreated 3xTg-AD animals (p < 0.05, n = 4) (Fig 4).

### Bexarotene treatment recovers impaired basal synaptic transmission in 3xTg-AD mice

**Fig 5:**
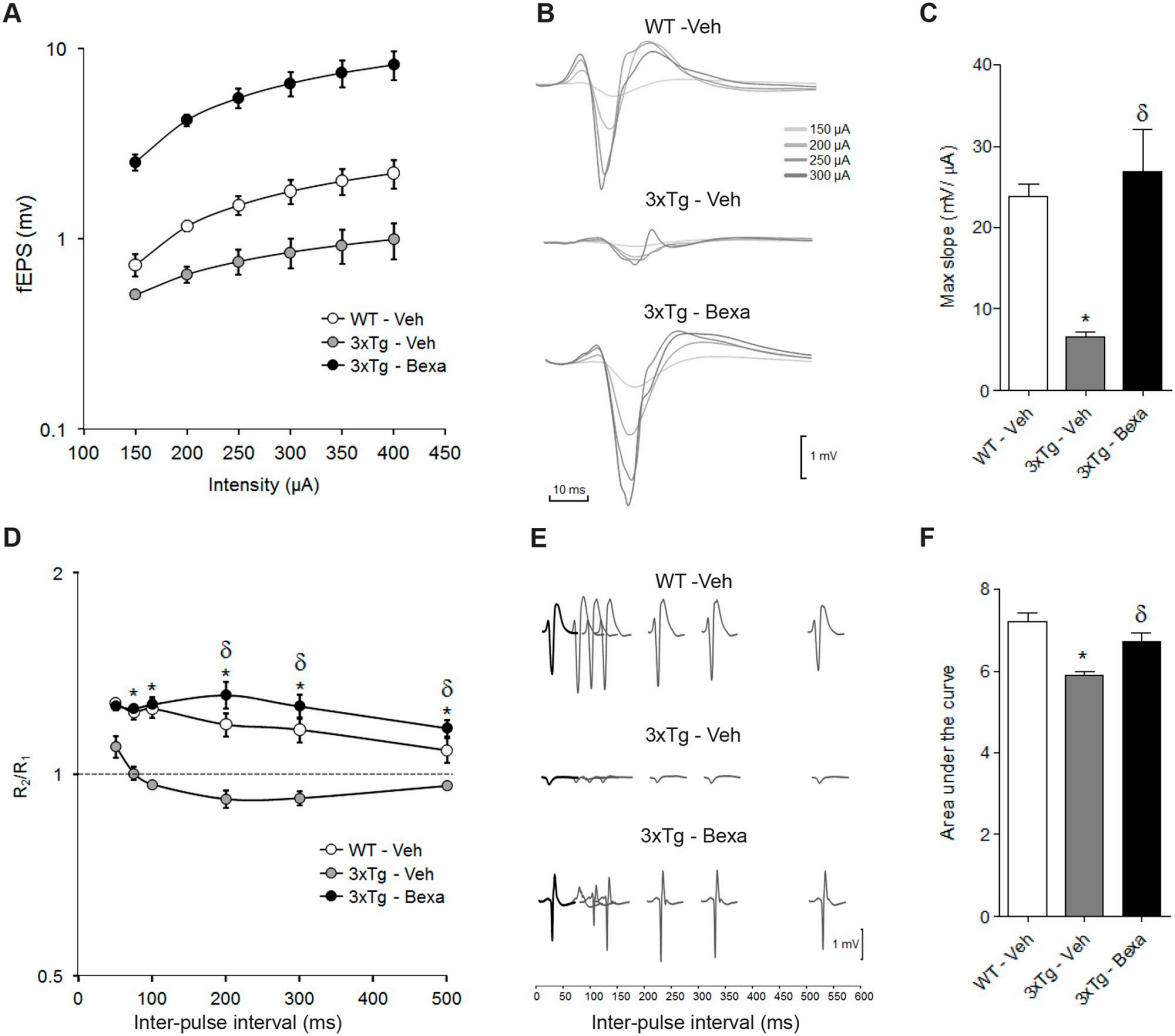
Bexarotene treatment rescued the deficits in basal synaptic transmission and paired pulse plasticity impairment at the CA3→CA1 commissural pathway in very old 3xTg-AD mice. **(A)** I/O curves for each experimental group respecting CA1 postsynaptic response amplitude. **(B)** Overlapping traces obtained from representative subjects of each experimental group illustrating CA1 postsynaptic response change as a function of contralateral CA3 stimulus intensity (indicated by the gray-scale). **(C)** Bar graph comparing maximal slopes of the I/O curves of the experimental groups. **(D)** Curves illustrating paired-pulse ratio change as a function of ISI for each experimental group. **(E)** Traces obtained from representative subjects of each experimental group illustrating the change of CA1 postsynaptic response to a pulse (gray lines) administered at different ISI after an initial pulse (black lines). **(F)** Bar graph comparing each experimental group’s paired-pulse ratio function’s area under the curve. Mean ± standard error of the mean are illustrated in each plot. Symbols: *, significant differences between 3xTg-AD groups and the control group (WT-veh); δ, significant differences between the bexarotene- and vehicle-treated 3xTg-AD groups (3xTg-Bexa and 3xTg-Veh). p <0.01.

Since synaptic dysfunction has been proposed as one of the major underpinnings of cognitive deficits in AD patients and in murine models of AD [19, 30–33], the present experiment evaluated *in-vivo* basal synaptic transmission and synaptic plasticity in the commissural CA3-CA1 synapses to correlate the effects of Bexarotene treatment with improvement in cognition of very old 3xTg-AD mice. In all three experimental groups (WT, untreated and treated 3xTg-AD mice), it was found that electrical stimulation in CA3 evoked typical CA1 fEPSP patterns with amplitude growing larger as stimulus intensity increased. Nevertheless, the fEPSP increasing amplitude as a function of stimulus intensity was significantly different from one experimental group to another (Fig 5B). The equations which best fit response amplitudes (A_fEPSP_) as a function of stimulus intensity (i_ST_) and the corresponding coefficient of determination (r^2^) for each group were as follows:

$$WT(Veh):A_{fEPSP}=6.16Ln(i_{ST})-27.88;r^{2}=0.97$$

$$3xTg-AD(Veh):A_{fEPSP}=1.68Ln(i_{ST})-6.68;r^{2}=0.92$$

$$3xTg-AD(Bexa):A_{fEPSP}=6.95Ln(i_{ST})-31.82;r^{2}=0.95$$

The maximum slopes of these functions were used for comparisons among the groups. The maximum slope of the I/O function was significantly smaller for the 3xTg-AD group (6.5 ± 4.3 mV/mA) than for the control WT group (23.8 ± 9.0 mV/mA; t = 4.417, p < 0.001), while the contrast maximum slope for the bexarotene treated 3xTg-AD group (26.9 ± 32.4 mV/mA) was not significantly different from that of the control group (t = 0.790, p = 0.431), but was significantly larger than that of the untreated 3xTg-AD group (t = 5.207, p < 0.001) (Fig 5C).

### Bexarotene modifies paired-pulse plasticity in 3xTG-AD mice

Control WT subjects displayed clear-cut paired-pulse facilitation (PPF) since their paired-pulse ratios were significantly greater than one (F_(6, 209)_ = 6.803, p < 0.001) (Fig 5D and 5E) for every ISI from 50 to 300 ms (50 ms, t = 5,153, p < 0.001; 75 ms, t = 4.404, p < 0.001; 100 ms, t = 4.702, p < 0.001; 200 ms, t = 3.465, p < 0.001; 300 ms, t = 3.074, p < 0.001), but not for 500 ms (t = 1.600, p = 0.111) (Fig 5D). In contrast, in untreated 3xTg-AD animals the only ISI with PPF was 50 ms (Fig 5D and 5E) (t = 2.888, p < 0.01), while the remaining ISIs were not significantly different from unity. Nevertheless, there was a non-significant tendency towards paired-pulse depression (200 ms, t = 2.365, p = 0.225; 300 ms, t = 2.287, p = 0.256; 500 ms, t = 1.142, p = 0.255) (Fig 5D). Bexarotene-treated 3xTg-AD mice recovered PPF (F _(6, 209)_ = 5.798, p < 0.001) (Fig 5D and 5E) in all ISIs (50 ms, t =, p =; 100 ms, t =, p =; 200 ms, t =, p =; 300 ms, t =, p =; 500 ms, t =, p =). The area under the curve (AUC) of PPF as a function of ISI was used to compare paired-pulse plasticity among groups. Significant differences in AUC were found among groups (F _(2, 89)_ = 13.186, p < 0.001). *Post hoc* comparisons showed that the AUC for 3xTg–AD animals (5.87 ± 0.12 ms) was significantly smaller than that for the control WT subjects (7.21 ± 0.21, t = 5.075, p < 0.001), and smaller than that for bexarotene-treated 3xTg-AD animals (3.72 ± 0.21, t = 3.22, p = 0.002) (Fig 5F).

### Bexarotene rescues LTP abnormalities in commissural CA3-CA1 synapses in 3xTg-AD mice

**Fig 6:**
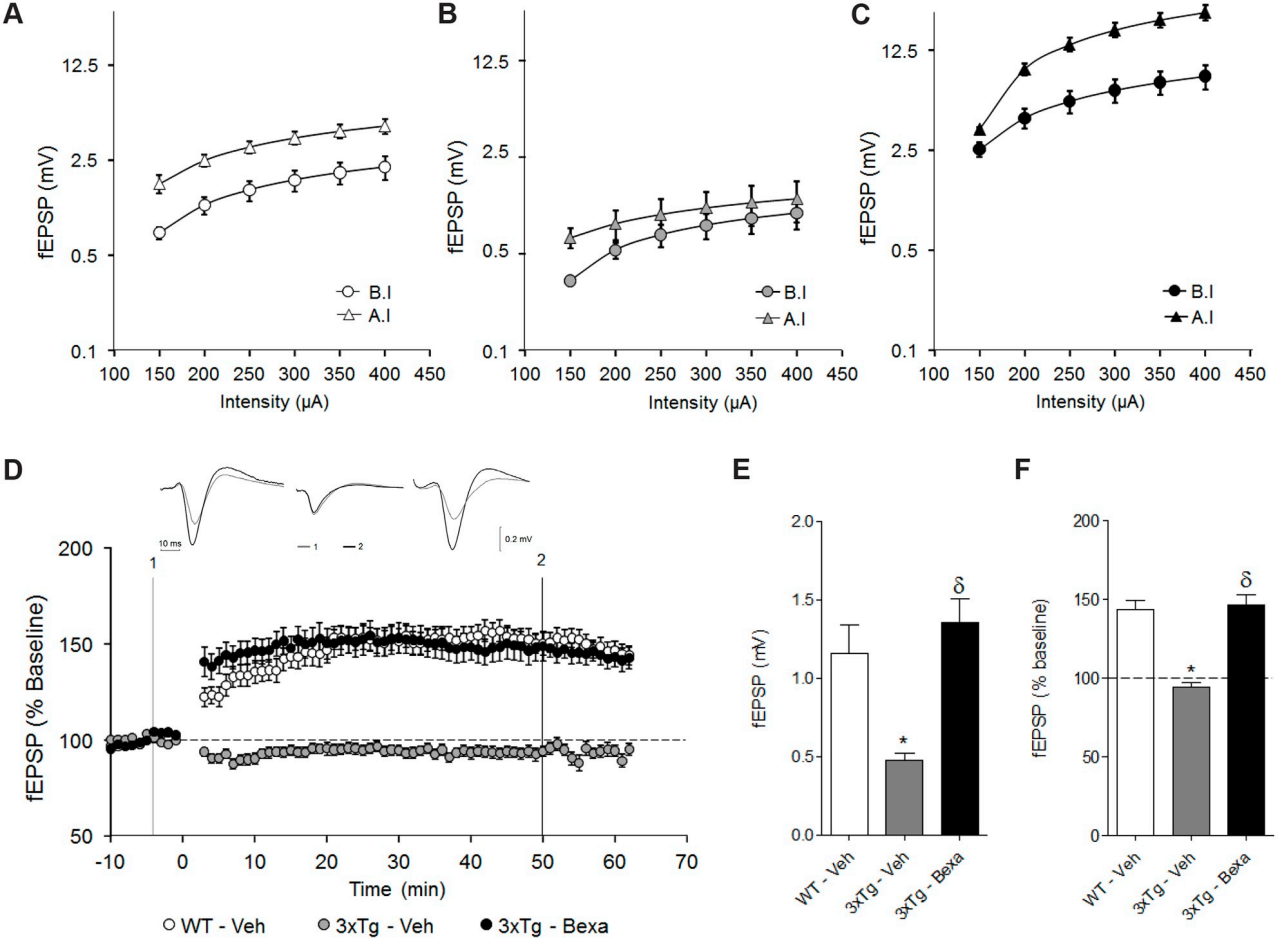
Bexarotene treatment rescued long-term potentiation impairment at the CA3→CA1 commissural pathway in very old 3xTg-AD mice. **A), B), and C)** I/O curves for each experimental group respecting CA1 postsynaptic response amplitude obtained either before (circles) or after (solid triangles) HFS. **(A)** WT-Veh. **(B)** 3xTg-Veh. **(C)** 3xTg-Bexa. **(D)** fEPSP amplitude (normalized respecting baseline) evolution before and after LTP-induction for each experimental group. HFS was administered at time zero. Before (1, gray traces) and after (2, black traces) upper traces of fEPSP were recorded. HFS was obtained from representative animals of each experimental group. **(E)** Bar graph comparing the mean fEPSP amplitude of each experimental group during baseline. **(F)** Bar graph comparing the relative fEPSP amplitude of each experimental group, as measured 50 min after LTP induction. Mean ± standard error of the mean are illustrated in each plot. Symbols: *, significant differences between 3xTg-AD groups and the control group (WT-Veh); δ, significant differences between the bexarotene- and vehicle-treated 3xTg-AD groups (3xTg-Bexa and 3xTg-Veh). p <0.01.

In control WT subjects, HFS induced a shift to the left in the I/O function, as evidenced by the I/O curve’s maximal slope becoming significantly steeper (t _(238)_ = -4.347, p < 0.001) after induction (A.I, 29.5 ± 11.15 mV/mA) than it was before induction (B.I, 23.8 ± 9.0 mV/mA) (Fig 6A). Bexarotene-treated 3xTg-AD subjects displayed a similar left shift of the I/O function after HFS, evidenced by a significantly steeper maximal slope of the I/O curve after induction (t _(398)_ = -3.431; p < 0.001) (45.7 ± 70.58 mV/mA) than before induction (26.89 ± 32.37 mV/mA) (Fig 6C).In contrast, HFS did not induce any significant shift in the I/O function of 3xTg-AD subjects, as evidenced by the lack of significant differences in maximal slope (B.I, 6.86 ± 5.75 mV/mA; A.I, 6.50 ± 4.32 mV/mA; t _(478)_ = -0.783; p = 0.434) (Fig 6B).

The fEPSP amplitude during baseline differed significantly between the 3xTg-AD group and the other two groups (F _(2, 107)_ = 14.696, p < 0.001). *Post hoc* comparisons demonstrated that the fEPSP amplitude (0.47 ± 0.23 mV) of 3xTg-AD subjects was significantly smaller than those of both the control WT group (1.16 ± 1.0 mV; t = 3.782, p< 0.001) and the bexarotene-treated 3xTg-AD group (1.36 ± 0.88 mV, t = 5.149, p < 0.001). There was no significant fEPSP amplitude differences (t = 1.074, p = 0.285) between the control WT group and the bexarotene-treated 3xTg-AD group (Fig 6E).

HFS induced significant fEPSP amplitude increases in the control WT subjects (F _(6, 209)_ = 9.613, p < 0.001) which persisted for at least 50 minutes (143.70 ± 30.35%, t = 6.014, p < 0.001) (Fig 6D and 6F). In contrast, no significant fEPSP amplitude changes were induced by HFS in 3xTg-AD mice (94.17 ± 19.22%) (F _(6, 293)_ = 1.146; p = 0.336) (Fig 6F). Interestingly, in bexarotene-treated 3xTg-AD mice, HFS induced a significant fEPSP amplitude increase (F _(6, 251)_ = 9.394; p < 0.001) (Fig 6D) which persisted for at least 50 minutes (146.17 ± 39.73%, t = 5.460, p <0.001). Fifty minutes after HFS, there were still significant fEPSP amplitude differences among groups (F _(2, 107)_ = 35.966, p < 0.001) (Fig 6F). *Post hoc* comparisons evidenced significantly smaller fEPSP amplitudes for 3xTg-AD subjects than those for the control WT subjects (t = 6.812; p < 0.001) and bexarotene-treated 3xTg-AD animals (t = 7.528; p < 0.001), while the fEPSP amplitudes of the latter two groups were the same (t = 0.329; p = 0.743).

### Bexarotene increases NeuN immunoreactivity associated with ApoE up-regulation in very old 3xTg-AD mice

**Fig 7:**
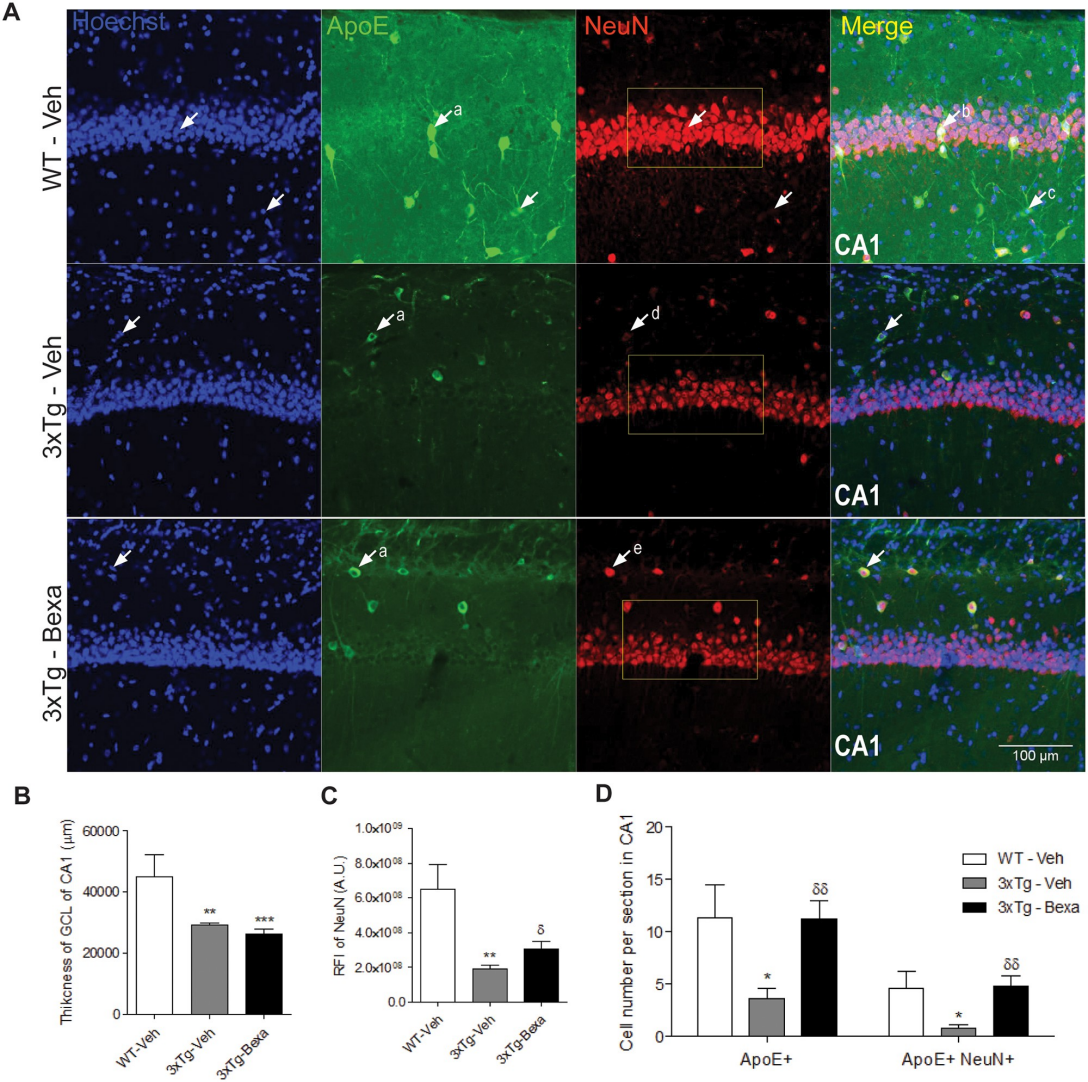
Bexarotene increases immunoreactivity of ApoE+ cells in soma associated NeuN expression in CA1 of the hippocampus. **(A)** Representative microphotographs of Hoechst (Blue), ApoE (Green) and NeuN (Red), immunofluorescence using confocal microscopy in CA1 of the hippocampus. **(B)** Quantitative analysis of thickness of the granular layer of NeuN+ cells in yellow box showing no significant differences between treated and untreated 3xTg-AD mice. **(C)** Quantitative analysis of RFI of NeuN in yellow box shows significantly greater RFI in treated 3xtg AD mice than in untreated 3xTg-AD mice. **(D)** Number of cells with ApoE overexpression in cell soma and with both ApoE overexpression and NeuN+ nuclei. Statistical analysis was performed by one-way ANOVA followed by the Bonferroni test. Data were expressed as mean ± S.E.M. Differences against control WT: *: p < 0.05, **: p < 0.01, ***: p < 0.001; and differences against untreated 3xTg-AD mice: ^δ^: p < 0.05, ^δδ^: p < 0.01; n = 4 per group.

In order to understand whether bexarotene changes CA1 neuron populations associated with improvement of synaptic plasticity and cognition, NeuN, a neuron marker primarily expressed in mature neurons, was evaluated by confocal microscopy [34] (Fig 7). To assess whether bexarotene increases the neuron maturation process, the experiment evaluated the thickness and expression of NeuN+ cells in the granular cell layer (GCL) of DG. GCL thickness was found to be significantly less in treated and untreated 3xTg-AD animals than in the control WT group (p < 0.005, p < 0.001) with no significant difference between GCL thicknesses of treated and untreated 3xTg-AD animals (Fig 7B). To evaluate NeuN expression, relative quantification of immunofluorescence intensity of DG in GCL was used (Yellow box in Fig 7A). Untreated 3xTg-AD mice were found to have significantly less intense immunofluorescence than that of the control WT group (p < 0.005). In addition, the bexarotene-treated 3xTg-AD group was found to have significantly more intense immunofluorescence than did the untreated 3xTg-AD group (p < 0.05) (Fig 7C).

Since it had already been shown that the ApoE expression levels are high in CA1 (Fig 3), it was still uncertain if somatic ApoE up-regulation (Fig 7Aa) is associated with increased NeuN expression. Two different cell populations with enhanced ApoE expression had been identified: one that had co-labelling of ApoE and NeuN (Fig 7Ab) and one with enhanced ApoE expression but without NeuN immunoreactivity (Fig 7Ac). A manual count of cells with high levels of ApoE expression in CA1 found that untreated 3xTg-AD animals had significantly less ApoE+ cells than did the control WT subjects regarding similar cells (p < 0.05) and that bexarotene recovered the number of ApoE+ cells in treated 3xTg-AD animals from the levels found in untreated 3xTg-AD animals (p < 0.05). When the population of NeuN+ co-labeled with ApoE cells among mature neurons was counted (Fig 7Ab), it was found that around 50% of high ApoE cells were mature neurons in the control WT mice. This proportion was significantly smaller in untreated 3xTg-AD animals (p < 0.005). Moreover; in contrast, it was found that bexarotene recovered the number of cells co-labeled for ApoE+ and NeuN+ in CA1 in treated 3xTg-AD animals.

## Discussion

Bexarotene, a selective RXR agonist which has been approved for treatment of cutaneous T-cell lymphoma by the FDA (US Food and Drug Administration) [35], has promise for treating Alzheimer’s disease because of its ability to improve cognition and reduce amyloid burden in murine models of AD [13]. The present study investigated potential molecular/functional mechanisms involved in the beneficial effects of Bexarotene in an AD mice model. Very old 3xTg-AD mice prone to suffering detrimental aging processes associated with amyloid deposits, tau pathology, neuroinflammation, synaptic transmission, plasticity impairment, and cognitive impairment were used as subjects since these characteristics are closely related to AD’s pathophysiology in humans [12, 19, 36, 37].

One month of bexarotene treatment improved learning, retention and ability to perform transference tasks in the hippocampal dependent MWM paradigm in very old 3xTg-AD mice, as measured by comparison with untreated 3xTg-AD mice. Previously, it had been shown that 90-day bexarotene treatment significantly improved the performance in the retention task in the MWM paradigm by 9-month-old APP/PS1 mice, but that 20-day-treatment of 7–8 month-old APP/PS1mice did not improve their performance in the same task [13]. In the same model, memory and cognition improvement was also found in 6-11-month-old mice (7-day-treatment) and 9-month-old mice (90-day-treatment) in a contextual fear-conditioning paradigm [21]. Another study of an APP/PS1 mice model found that both 7-day treatment of 6–11 month-old mice and 90-day treatment of 9-month old mice improved memory and cognition in a contextual fear-conditioning paradigm [21]. In addition, the same authors have also shown memory improvement in 7-month-old APP/PS1ΔE9 mice, irrespective of whether the mice expressed human APOE3 or APOE4 alleles (Fitz et al., 2013).

It was also observed that bexarotene increased ApoE protein expression in CA1. ApoE is a direct target gene regulated either alone or synergistically by LXRs and PPARγ [2, 38];[15]. Under the conditions in the present study, ApoE expression was significantly increased only at CA1 in treated 3xTg-AD animals. No significant changes were found in other hippocampal regions or in the cerebral cortex. Similar data for LXR agonist treatment have previously been reported [2, 5, 39].

Despite the absence of significant observable changes in amyloid deposits, there was a significant reduction in the amount of soluble Aβ (x-42) in the cortex but not in the hippocampus. This indicates that cognitive improvement is independent of amyloid pathology changes in the hippocampus. However, it has been shown that cortical damage causes impairment of MWM performance (D’Hooge and De Deyn, 2001). Despite the fact that functional studies have demonstrated that ApoE expression induced by RXRs:LXRs or RXRs:PPARγ contributes to acceleration of β-amyloid clearance (Cramer et al., 2012;Jiang et al., 2008;Escribano et al., 2010), under the experimental conditions in the present study, there were no apparent associations between amyloid clearance and ApoE overexpression in regions as CA1 or between amyloid clearance and soluble β-amyloid reduction in the cortex. Therefore, β-amyloid regulation appears to be independent of ApoE up-regulation in the model herein proposed.

Another important finding was that the fact that bexarotene-induced ApoE up-regulation in CA1 of 3xTg-AD animals is probably associated with cognitive improvement in MWM tests. A recent study that used ApoE-KO mice demonstrated that increased ApoE expression mediated by bexarotene is necessary for reversion of traumatic brain injury-induced memory and learning impairment [40]. Even though astrocytes are the primary source of ApoE [41], the present study has shown that the somata of mature neurons (NeuN+ cells) also express large amounts of ApoE, as demonstrated by ApoE and NeuN co-localization.

Expression level and localization of NeuN have been associated with neuronal health, so high levels of NeuN expression characterize healthy neurons, while decreased expression indicates degeneration of differentiated neurons [42–44]. High levels of NeuN expression were observed in the control WT animals while greater numbers of ApoE+, and NeuN+ co-labeled cells were observed in greater amounts in bexarotene-treated 3xTg-AD mice than in untreated 3xTg-AD mice (Fig 7Ad–7Ae). ApoE has been associated with neuronal injury [6, 45]. In the model herein presented, expression changes in ApoE may be caused by the aging process itself and by the presence of abnormal tau and acute amyloid pathology characteristic of the model [46, 47]. NeuN up-regulation in bexarotene-treated 3xTg-AD mice indicates that bexarotene is mediating neuronal protection signaling most probably mediated by ApoE or by the control of other currently unknown pathways.

Bexarotene was found to have a preponderant, general anti-inflammatory effect through decreasing immunoreactivity of GFAP and Iba1 in the cortex and hippocampus. As previously reported, patterns of activated microglia and astrogliosis, which were not apparent in WT controls, were observed in 3xTG-AD mice (Caruso et al., 2013): the amount and areas affected by both of these morphological patterns were significantly reduced by treatment with bexarotene.

The potent anti-inflammatory effect of bexarotene observed in treated 3xTg-AD mice may be associated with the activation of PPARs::RXRs and/or LXRs::RXR heterodimers, which have previously been described as having potent anti-inflammatory effects on the brain [2, 28, 29, 48].

Finally, bexarotene improved not only basal synaptic transmission, but also short- and long-term synaptic plasticity. Basal synaptic transmission impairment in untreated 3xTg-AD mice, indicated here by I/O curves that are significantly smaller than those of wild-type mice, is in agreement with what was previously described in young [19] and old (Sacheti et al.2013) 3xTg-AD animals. Bexarotene-treated 3xTg-AD mice displayed I/O curves similar to those of wild-type mice, indicating restoration of basal synaptic transmission.

Unlike the robust PPF found in control wild-type animals, very old 3xTg-AD mice displayed impaired PPF (100 to 500-ms ISI). In contrast, PPF in bexarotene-treated 3xTg-AD subjects was not different than that found in control subjects. This suggests that the bexarotene treatment reverted PPF impairment in 3xTg-AD mice. It has been reported that both young and old 3xTg-AD mice have displayed increased PPF in CA1 [49], but those apparently contradictory findings were obtained by stimulating temporoammonic inputs to CA1. It has also been reported that the PPF of very young 3xTg-AD mice is similar to that in CA3 to CA1 synapses of wild-type mice due to abnormally enhanced expression of presynaptic ryanodine receptors [49, 50]. Another study reported that both young and old 3xTg-AD mice display abnormally low PPF in CA3 to CA1 synapses [51].

The induction treatment used in the present study was not efficient to elicit significant LTP in 3xTg-AD mice. This finding is in agreement with previous reports evaluating CA3-CA1 synaptic plasticity in hippocampal slices from 3xTg-AD mice [50]. However, both bexarotene-treated 3xTg-AD and wild-type mice displayed robust LTP after stimulation was induced, which indicates that bexarotene also reverts LTP impairment in 3xTg-AD animals. The mechanism involved in this case of synaptic improvement is most probably associated with neuronal recovery of dysfunctional neurons in the hippocampus and is probably associated with increased ApoE expression and signaling. In a recent report, our group has shown that LXR agonist GW3965 enhanced synaptic plasticity through a protein synthesis-dependent mechanism [2]. In addition, GW3965 in elderly 3xTg-AD mice induced hypomethylation in gene promoters of synaptic function-related genes, including Synapsin 1 (Syn 1) and Synaptophysin (Syp) [52]. More recently, it has been shown that expression of NR1, GluR1, PSD95 and SYP proteins increased the hippocampi and cerebral cortices of bexarotene-treated 20 to 24-month-old mice [53]. Moreover, it was shown that bexarotene improves dendritic morphology and complexity in CA1, even in an APOE4 context [16]. Changes in transcription observed under the bexarotene treatment in the present murine model seem to be mediated by LXRs::RXRs activation [2, 54–56].

Taken together, the results of this study suggest that bexarotene treatment leads to a number of molecular and morphological changes in very old 3xTg-AD mice, and an association between the expression of ApoE, neuronal protection and functional improvement has been found. However, the role of APOE in specific areas of the hippocampus remains to be clarified. It is well known that interaction of ApoE with its receptors may affect diverse signaling pathways that modulate synaptic plasticity, dendritic spine development, neurite growth, regulation of neuronal migration, and cellular transport [57, 58]. In addition, the results of the present study further support the idea of a predominantly anti-inflammatory role for bexarotene in very old 3xTg-AD mice. This effect could be required for the beneficial effects of this RXR agonist and may precede the recovery of synaptic functioning through modulation of synaptic-related protein synthesis in the hippocampus. For these reasons, bexarotene and similarly acting pharmaceuticals may become therapeutic alternatives for Alzheimer’s disease.

## Supporting information

S1 File Experimental preparation for electrophysiological experiments and electrode placement. **(A)** Schematic representation of the dorsal view of a mouse head and brain indicating stimulation (STM) and recording (REC) electrode insertion points. **(B)** Summary of the final placement of the stimulating (stars) and recording electrodes determined by electrolytic lesions on the Bregma -2 mm coronal section of mouse brain [59]. Blue symbols represent bexarotene-treated animals, red symbols represent vehicle-treated mice. **(C)** Dark-field micrographs of electrolytic lesions caused through the point of the recording (left) and stimulating (right) electrodes at their final placement.
(PDF)

S2 File Bexarotene effects over GFAP and ApoE in treated 3xTg-AD mice. **(A)** Representative microphotographs of GFAP (Red), ApoE (Green) immunofluorescence and Hoechst (Blue) using confocal microscopy. **(B)** Quantitative analysis of ApoE immunoreactivity of data presented in A indicating significant increases of RFI of ApoE in CA1 of the hippocampus produced by Bexarotene in treated 3xTg-AD mice. **(C)** Quantitative analysis of GFAP immunoreactivity of data presented in A indicating a significant reduction of astrogliosis produced by Bexarotene in treated 3xTg-AD mice. Statistical analysis was performed by one-way ANOVA followed by the Bonferroni test. Data were expressed as mean ± S.E.M. Differences against control WT: *: p < 0.05, **: p < 0.01; and differences against untreated 3xTg-AD: ^δ^: p < 0.05, ^δδ^: p < 0.01, ^δδδ^: p < 0.001; n = 4 per group.
(PDF)

S3 File Bexarotene effect in Tau pathology in treated 3xTg-AD mice. **(A)** Representative microphotographs of Tau (Green) immunofluorescence in CA1 and CA3 of the hippocampus. **(B)** Quantitative analysis of the area of Tau immunoreactivity in CA1. **(C)** Quantitative analysis of the area of Tau immunoreactivity in CA3. Statistical analysis was performed by one-way ANOVA followed by Bonferroni testing. Data are expressed as mean ± S.E.M. Differences against control WT: *: p < 0.05 n = 4 per group.
(PDF)
