# Supplementary material for: Bexarotene therapy ameliorates behavioral deficits and induces functional and molecular changes in very-old Triple Transgenic Mice model of Alzheimer´s disease
Source: PLoS One. 2019 Oct 9;14(10):e0223578. doi: 10.1371/journal.pone.0223578 (PMC6785083; doi:10.1371/journal.pone.0223578)
Supplement: S1 File — (A) Schematic representation of the dorsal view of a mouse head and brain indicating stimulation (STM) and recording (REC) electrode insertion points. (B) Summary of the final placement of the stimulating (stars) and recording electrodes determined by electrolytic lesions on the Bregma -2 mm coronal section of mouse brain [59]. Blue symbols represent bexarotene-treated animals, red symbols represent vehicle-treated mice. (C) Dark-field micrographs of electrolytic lesions caused through the point of the recording (left) and stimulating (right) electrodes at their final placement. (PDF) [file pone.0223578.s001.pdf]

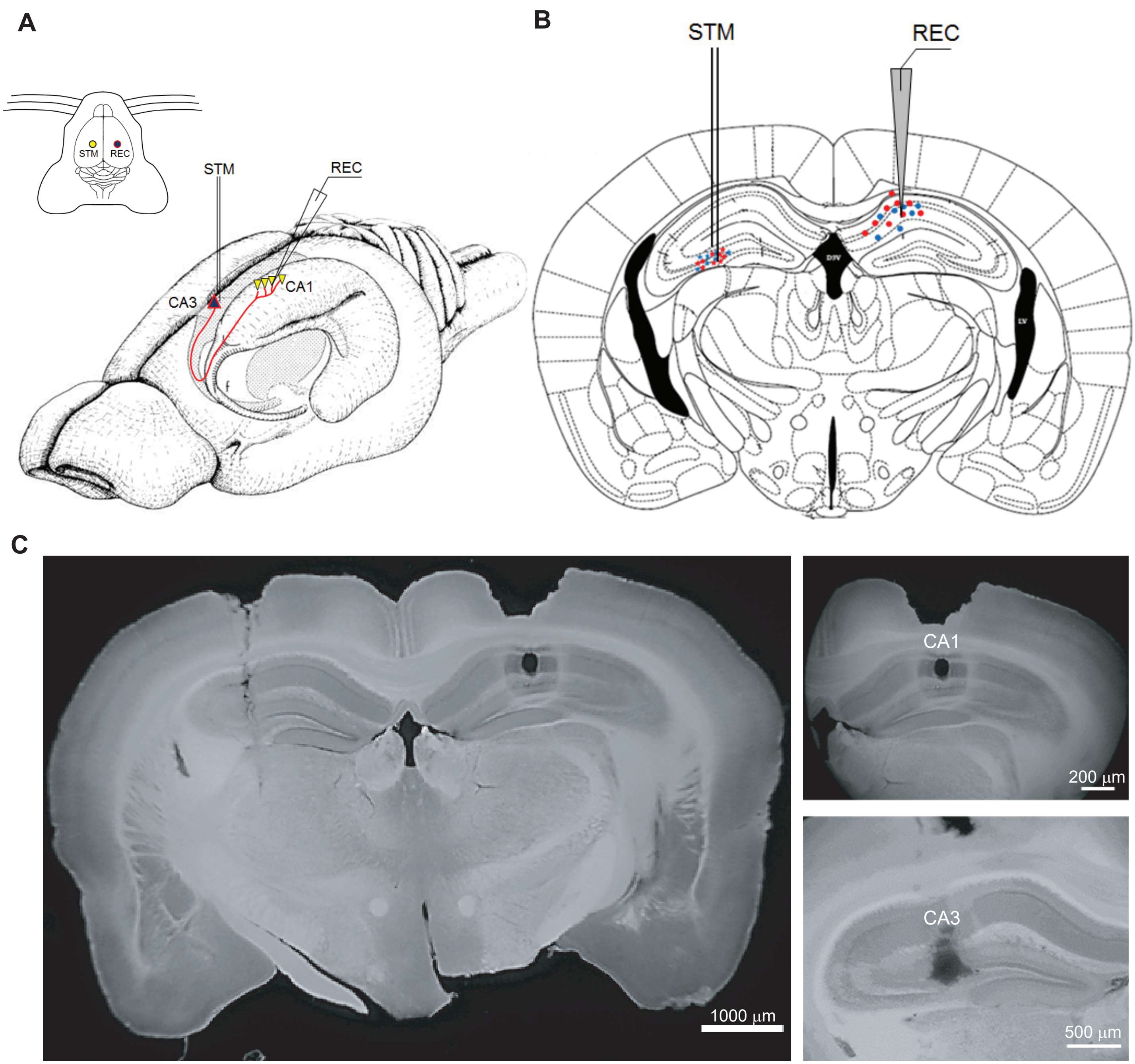


**Figure S1 Experimental preparation for electrophysiological experiments  and electrode placement. (A)** Schematic representation of the dorsal view of a mouse head and brain indicating stimulation (STM) and recording (REC) electrode insertion points. (**B)** Summary of the final placement of the stimulating (stars) and recording electrodes determined by electrolytic lesions on the Bregma -2 mm coronal section of mouse brain [53]. Blue symbols represent bexarotene-treated animals, red symbols represent vehicle-treated mice. **(C)** Dark-field micrographs of electrolytic lesions caused through the point of the recording (left) and stimulating (right) electrodes at their final placement.
