# Supplementary material for: Bexarotene therapy ameliorates behavioral deficits and induces functional and molecular changes in very-old Triple Transgenic Mice model of Alzheimer´s disease
Source: PLoS One. 2019 Oct 9;14(10):e0223578. doi: 10.1371/journal.pone.0223578 (PMC6785083; doi:10.1371/journal.pone.0223578)
Supplement: S2 File — (A) Representative microphotographs of GFAP (Red), ApoE (Green) immunofluorescence and Hoechst (Blue) using confocal microscopy. (B) Quantitative analysis of ApoE immunoreactivity of data presented in A indicating significant increases of RFI of ApoE in CA1 of the hippocampus produced by Bexarotene in treated 3xTg-AD mice. (C) Quantitative analysis of GFAP immunoreactivity of data presented in A indicating a significant reduction of astrogliosis produced by Bexarotene in treated 3xTg-AD mice. Statistical analysis was performed by one-way ANOVA followed by the Bonferroni test. Data were expressed as mean ± S.E.M. Differences against control WT: *: p < 0.05, **: p < 0.01; and differences against untreated 3xTg-AD: δ: p < 0.05, δδ: p < 0.01, δδδ: p < 0.001; n = 4 per group. (PDF) [file pone.0223578.s002.pdf]

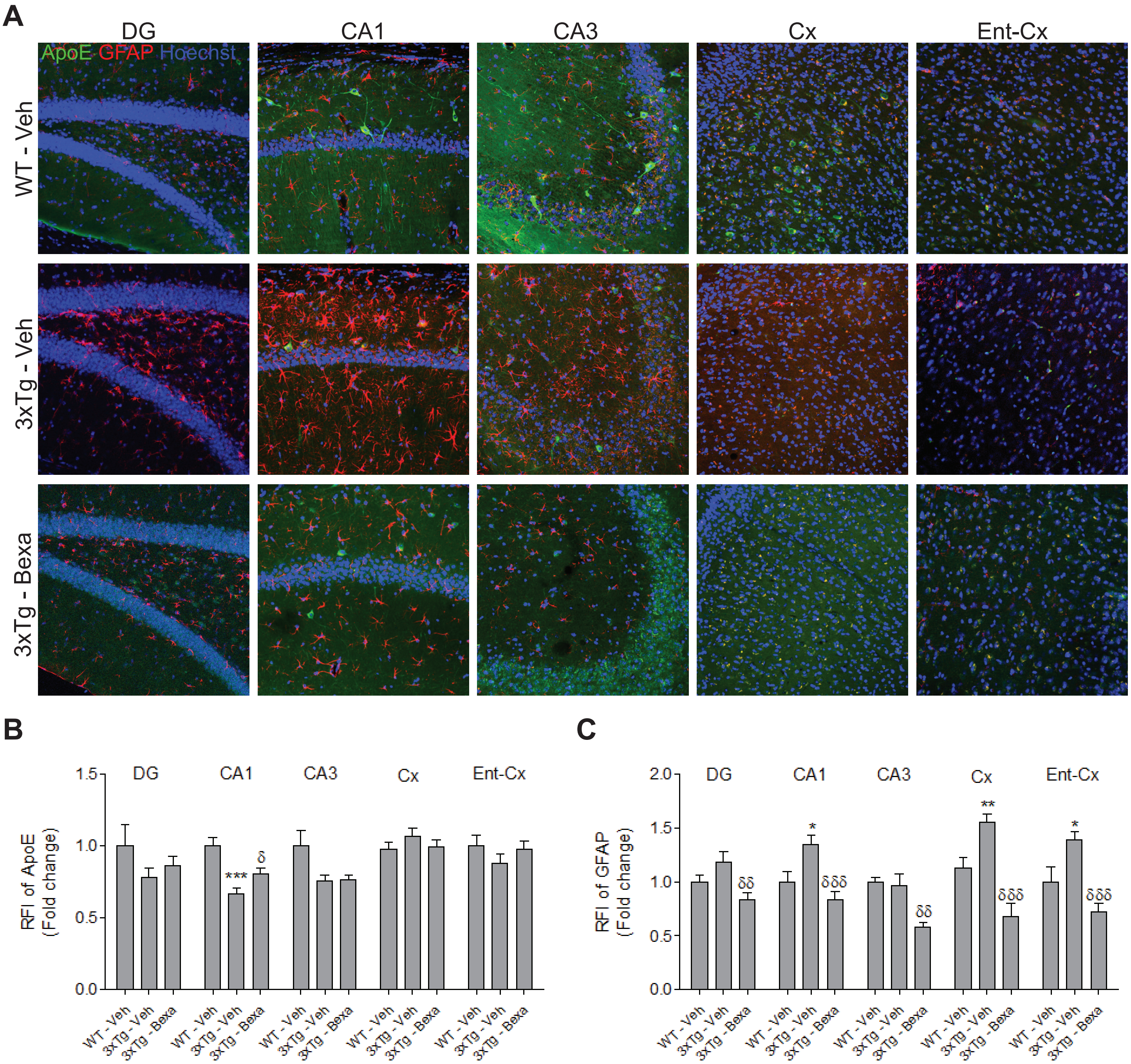

Figure S2. Bexarotene effects over GFAP and ApoE in treated 3xTg-AD mice. (A) Representative microphotographs of GFAP (Red), ApoE (Green) immunofluorescence and Hoechst (Blue) using confocal microscopy. **(B)** Quantitative analysis of the ApoE immunoreactivity of data presented in A indicate significant increases of RFI of ApoE in CA1 of the hippocampus produced by Bexarotene in treated 3xTg-AD mice. **(C)** Quantitative analysis of GFAP immunoreactivity of data presented in A indicating significant reduction of astrogliosis produced by Bexarotene in treated 3xTg-AD mice. Statistical analysis was performed by one-way ANOVA followed by Bonferroni testing. Data are expressed as mean ± S.E.M. Differences against control WT: *: $p < 0.05$, **: $p < 0.01$; and differences against untreated 3xTg-AD: δ: $p < 0.05$, δδ: $p < 0.01$, δδδ: $p < 0.001$; n = 4 per group.
