# Supplementary material for: Bexarotene therapy ameliorates behavioral deficits and induces functional and molecular changes in very-old Triple Transgenic Mice model of Alzheimer´s disease
Source: PLoS One. 2019 Oct 9;14(10):e0223578. doi: 10.1371/journal.pone.0223578 (PMC6785083; doi:10.1371/journal.pone.0223578)
Supplement: S3 File — (A) Representative microphotographs of Tau (Green) immunofluorescence in CA1 and CA3 of the hippocampus. (B) Quantitative analysis of the area of Tau immunoreactivity in CA1. (C) Quantitative analysis of the area of Tau immunoreactivity in CA3. Statistical analysis was performed by one-way ANOVA followed by Bonferroni testing. Data are expressed as mean ± S.E.M. Differences against control WT: *: p < 0.05 n = 4 per group. (PDF) [file pone.0223578.s003.pdf]

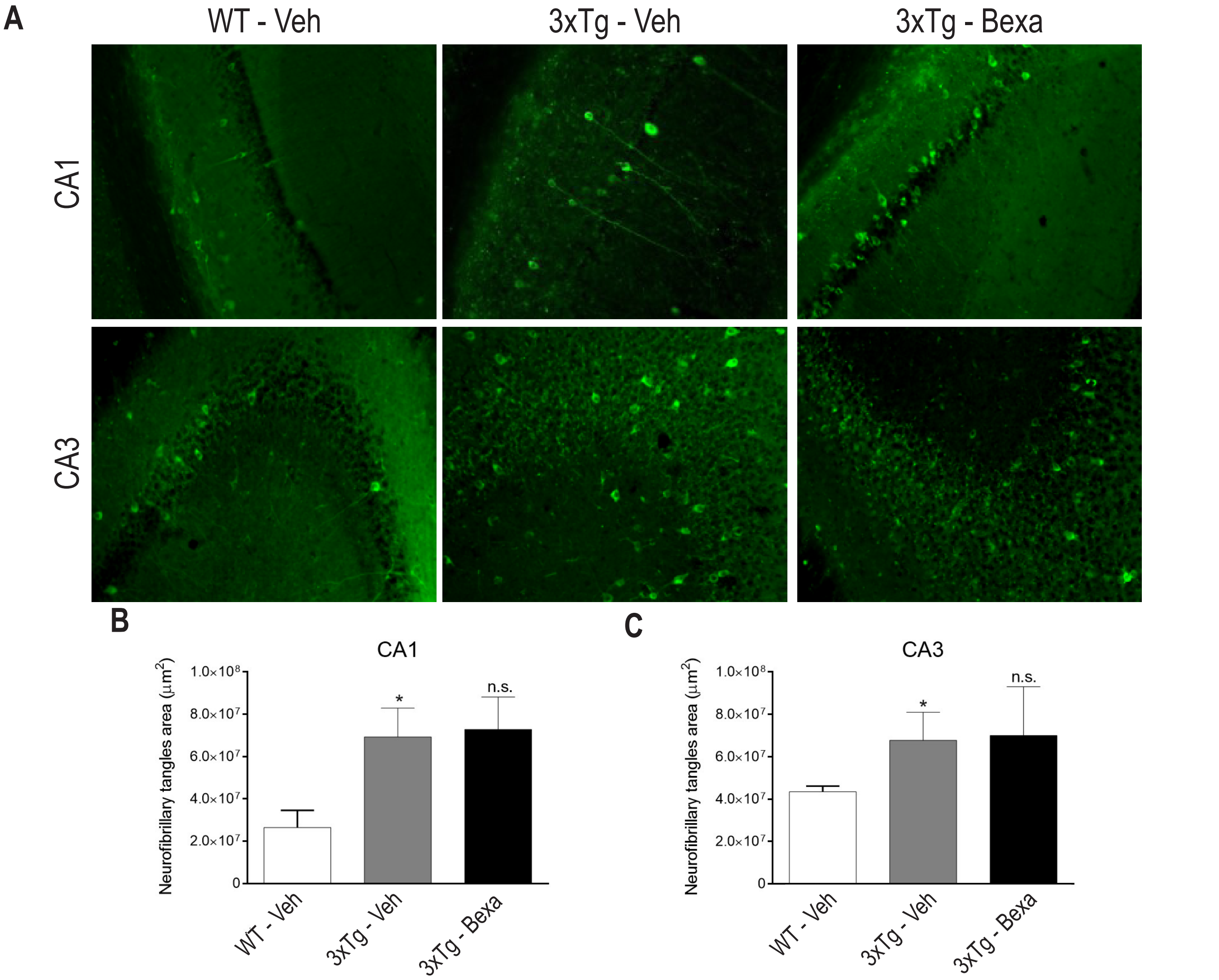

Figure S3. Bexarotene effect in Tau pathology in treated 3xTg-AD mice. (A) Representative microphotographs of Tau (Green) immunofluorescence in CA1 and CA3 of the hippocampus. (B) Quantitative analysis of the area of Tau immunoreactivity in CA1. (C) Quantitative analysis of the area of Tau immunoreactivity in CA3. Statistical analysis was performed by one-way ANOVA followed by Bonferroni testing. Data are expressed as mean ± S.E.M. Differences against control WT: *: $p < 0.05$ $n = 4$ per group.
